# Active Immunization Against hIAPP Oligomers Ameliorates the Diabetes- Associated Phenotype in a Transgenic Mice Model

**DOI:** 10.1038/s41598-017-14311-1

**Published:** 2017-10-25

**Authors:** Yaron Bram, Sivan Peled, Sayanti Brahmachari, Michael Harlev, Ehud Gazit

**Affiliations:** 1 Department of Molecular Microbiology and Biotechnology, Tel Aviv University, Ramat Aviv, Tel Aviv, 69978 Israel; 2 Faculty of Medicine, Tel Aviv University, Ramat Aviv, Tel Aviv, 69978 Israel; 3 Present Address: Weill Cornell Medicine, Cornell University, New York, 10021 USA

## Abstract

Type 2 diabetes is characterized by insulin tolerance in target cells followed by a reduction of pancreatic β-cell mass. Islet amyloid polypeptide oligomeric assemblies were shown to contribute to β-cell apoptosis by forming discrete pores that destabilize the cellular membrane. We previously characterized α-helical cytotoxic islet amyloid polypeptide oligomers which interact with cell membranes, following a complete internalization that leads to cellular apoptosis. Moreover, antibodies which bind the oligomers and neutralize the cytotoxicity were exclusively identified in the serum of type 2 diabetes patients. Here, we examined the usage of the newly characterized oligomers as an active immunization agent targeting amyloid self- assembly in a diabetes-associated phenotype transgenic mice model. Immunized transgenic mice showed an increase in hIAPP-antibody serum titer as well as improvement in diabetes-associated parameters. Lower fasting blood glucose levels, higher insulin, and lower islet amyloid polypeptide accumulation were observed. Furthermore, antibodies derived from the immunized mice reduced hIAPP oligomers cytotoxicity towards β-cells in a dose-dependent manner. This study highlights the significance of targeting the early amyloid self-assembly events for potential disease management. Furthermore, it demonstrates that α-helical oligomers conformers are valid epitope for the development of future immunization therapy.

## Introduction

Diabetes mellitus (DM) is one of the most common non-communicable diseases globally. According to the International Diabetes Federation (IDF), 387 million people suffered from diabetes in 2014 which resulted in 4.9 million deaths; by 2035, this figure is expected to rise to 592 million. Type 2 diabetes mellitus (T2DM) is a chronic syndrome that occurs when there is a decrease in the response of target cells towards insulin signaling (“insulin resistance”), leading to an increasing demand for insulin secretion^1^. In addition, T2DM is accompanied by a reduction in β-cell mass and function over time^2^.

Islet amyloid polypeptide (IAPP) aggregation was suggested as one of the factors which contribute to pancreatic deterioration^3,4^. IAPP is one of the major hormones produced by pancreatic β-cells. It is co-stored and secreted along with insulin via vesicles and plays a role in gastric emptying and glucose homeostasis^5^. The appearance of amyloid aggregates in the pancreatic tissue of diabetic patients was first reported more than a century ago^6^. These aggregates were later successfully isolated and IAPP was identified as the key component^7,8^. Similar to other amyloid-associated diseases, the appearance of fibrillar deposits in post-mortem histological sections of damaged organs led to the assumption that they play a critical role in tissue degeneration^9^. However, in later studies a poor correlation between the number of amyloid aggregates and disease severity was observed^10^; furthermore, instances of cell death was detected prior to amyloid fibril formation^11^. Studies for other pathological components led to the recognition of diffusible amyloid oligomers and their important involvement in disease etiology^12–16^. It has been well established that amyloidogenic peptides cause membrane perturbation, and in specific, amyloid oligomers increase lipid bilayer permeability regardless of their sequence, by establishing discrete pores^17–20^. In contrast, fibrils and soluble low molecular weight species showed no detectable effect on membranes^21,22^.

Previously, we characterized human IAPP (hIAPP) soluble oligomers, with size distribution from monomers up to 90 kDa conformers. These oligomers have a predominantly α-helical conformation, consistent with studies by others showing the transition of an IAPP from its unfolded random coil state to α-helical conformation, upon interaction with different membranes^23^. Our findings indicate that these α-helical assemblies attach to the cellular membrane, form discrete pores that result in apoptosis and eventually cell death. Furthermore, to examine the relevance of the oligomers formed *in vitro* to the etiology of the disease, we detected the presence of antibodies that recognize the oligomeric conformers in the sera of T2DM patients. These antibodies showed considerably higher binding toward oligomeric assemblies and significantly less affinity toward amyloid fibrils or monomeric hIAPP. Interestingly, these antibodies were able to appreciably reduce the toxicity of hIAPP oligomers towards pancreatic cells in a dose-dependent manner, suggesting a direct involvement of hIAPP in the disease pathology. This finding also suggested that hIAPP oligomers have potential to be used as active immunization agents against hIAPP self-assembly cascade. To evaluate this, we employed a transgenic mice model [FVB/N-Tg(Ins2-IAPP)RHFSoel/J] expressing hIAPP under the regulatory control of the rat insulin II promoter^10^. Homozygous males spontaneously developed symptoms associated with T2DM, such as β-cell apoptosis, high blood glucose levels, insulin reduction and hIAPP deposits.

**Figure 1:**
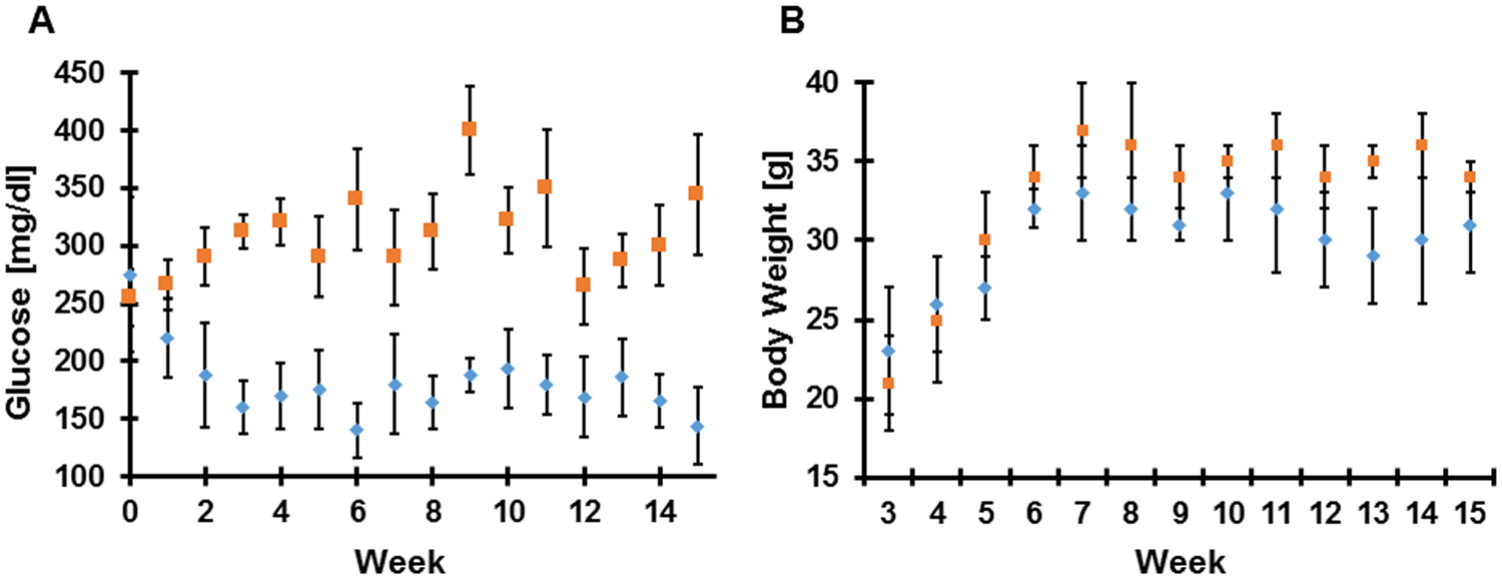
Immunization with hIAPP oligomer reduces the T2DM phenotype. (**A**) Mice received intraperitoneal injections of hIAPP oligomers (blue diamond) or vehicle (red squares), along with an adjuvant. Blood glucose levels were evaluated every 7 days. As shown, there was a statistically significant difference between the mock and treated groups (N = 13 in each group, values are means ± s.d). (**B**) Body weight measurements over time in mice receiving vehicle (red squares) and treatment (blue diamond, N = 10 in each group).

To evaluate the effect of immunization, homozygous males were intraperitoneally injected with hIAPP oligomers emulsified with Freund’s adjuvant (see Methods section) starting at 3 weeks of age, prior to the appearance of hIAPP pancreatic deposits at 8 weeks of age. An additional boost was given 2 weeks after the first injection, and monthly thereafter. The treated group was compared to a mock group treated only with the vehicle (N = 13, each group). Blood glucose level was measured every 5 days. As shown in Fig. 1A, we observed significant differences between the two groups, this remained consistent throughout the entire experiment. While blood glucose levels were above 230 mg/dL in the mock group, the treated group remained below 190 mg/dL for most of the experiment, the baseline fasting glucose levels of FVB/N male mice ranged from 110–190 mg/dL (data not shown). Furthermore, we observed that the treated group was slightly leaner than the control group (Fig. 1B).

**Figure 2:**
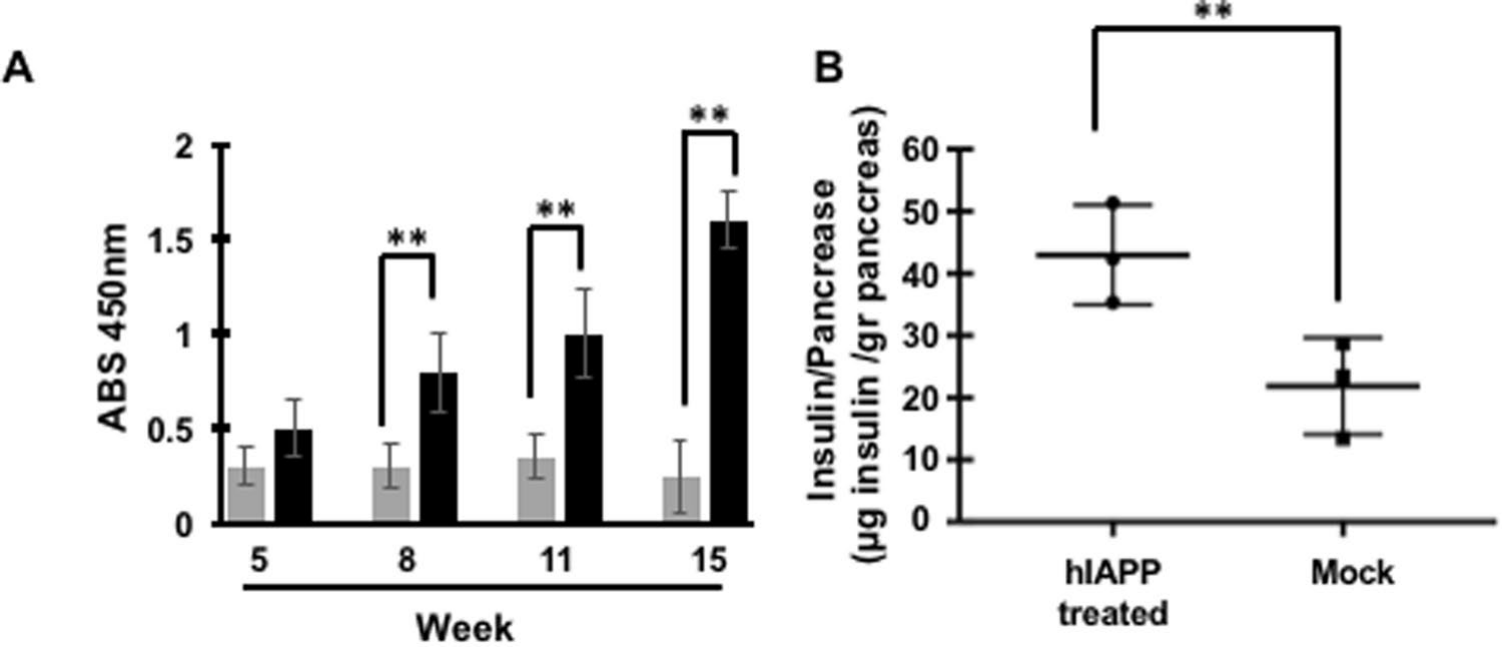
Treated mice developed neutralizing antibodies that delayed β-cell loss. (**A**) Blood samples were collected by submandibular bleeding. α-hIAPP antibody serum titer was evaluated by ELISA assay (N = 13, sera dilution 1:500, values are means ± s.d., Student’s t-test **P < 0.005). Grey bars denotes the mock treatment and the black bar denotes the hIAPP treated group. (**B**) At the experiment endpoint, three mice from each group were chosen randomly. The pancreas was removed and total insulin content per tissue weight was determined (data point values are presented).

**Figure 3:**
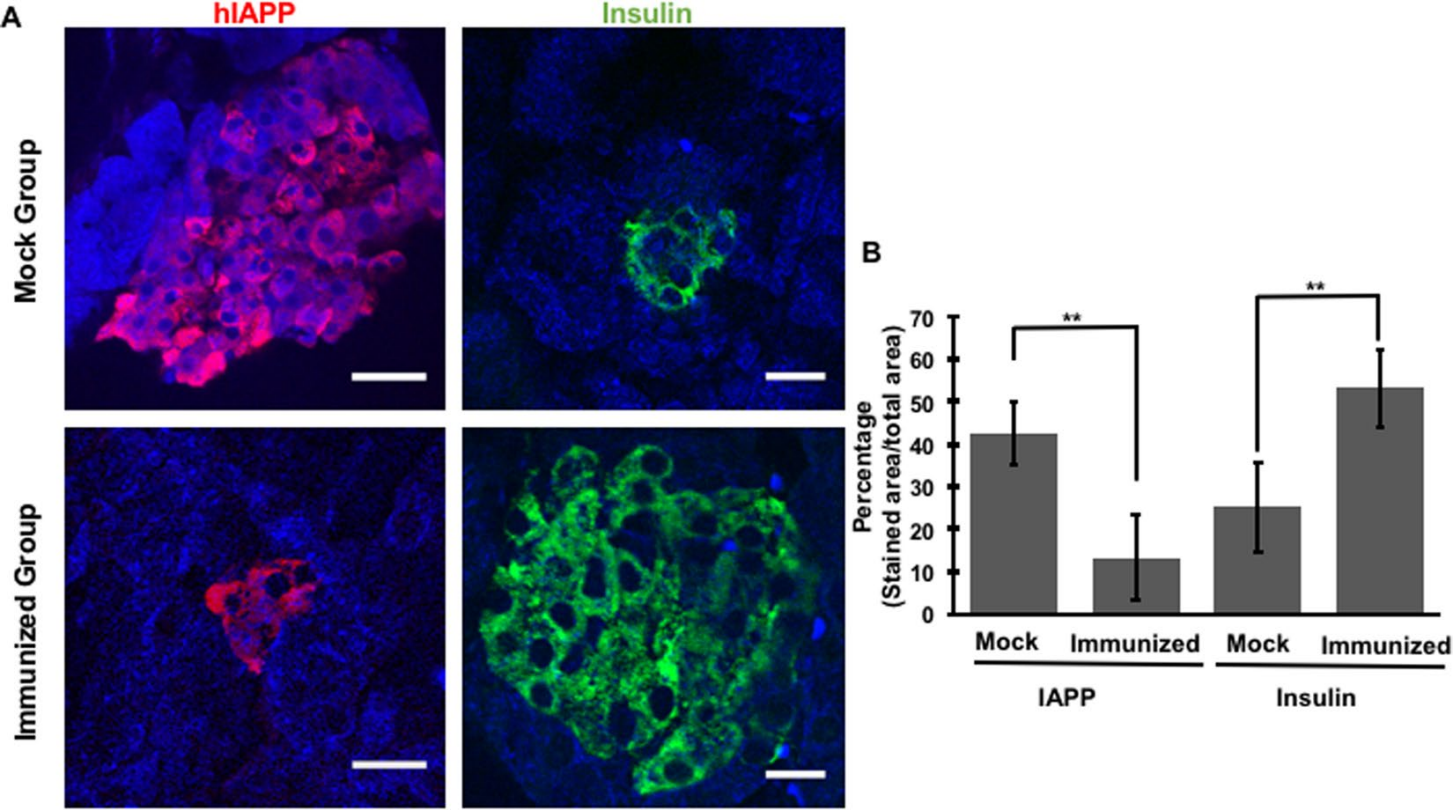
Treated mice had higher insulin content and less hIAPP accumulation in the pancreas. **(A)** Confocal microscopy analysis of histological sections of treated mice compared with the mock group. Histological sections were stained for hIAPP (red) and insulin (green). As shown, the treated group showed higher insulin levels and lower levels of hIAPP accumulation. Scale bar 200 µm. (**B**) Quantification of insulin and hIAPP in immunized and mock groups (N = 4 per group), **P < 0.005.

Blood samples were collected every 2 weeks by submandibular bleeding and the hIAPP antibody titer was determined by ELISA assay. As shown in Fig. 2A, vaccination with hIAPP oligomers induced hIAPP antibody production. We observed a steady increase in antibody levels from 5 weeks of age reaching a titer maximum at 11 weeks of age. Furthermore, the mock group did not show an increase in hIAPP antibodies titer. Transgenic homozygous males display a reduction in insulin secretion, despite severe hyperglycemia as a result of hIAPP accumulation that leads to a decrease in β-cells. To estimate the impact of immunization on the survival of functional β-cells in the pancreas, mice were sacrificed at 15 weeks of age and the pancreas was isolated to determine the insulin quantity in the tissue. At the experimental endpoint, the treated group had significantly higher amounts of insulin compared to the mock group (Fig. 2B). Immunostaining of pancreatic sections validated these results, showing larger insulin positive area and smaller hIAPP positive area (Fig. 3A). Immunization with hIAPP oligomers significantly increased insulin content by ∼2 fold and decreased hIAPP levels by ∼3 fold (Fig. 3B). These results, along with the reduction in blood glucose levels and an increase in the hIAPP antibody titer, suggest that immunization with low molecular weight hIAPP oligomers may lead to an increase in neutralizing antibodies that are able to inhibit the self-assembly cascade in its earliest steps, decreasing hIAPP-associated cytotoxicity and reducing β-cell loss.

**Figure 4:**
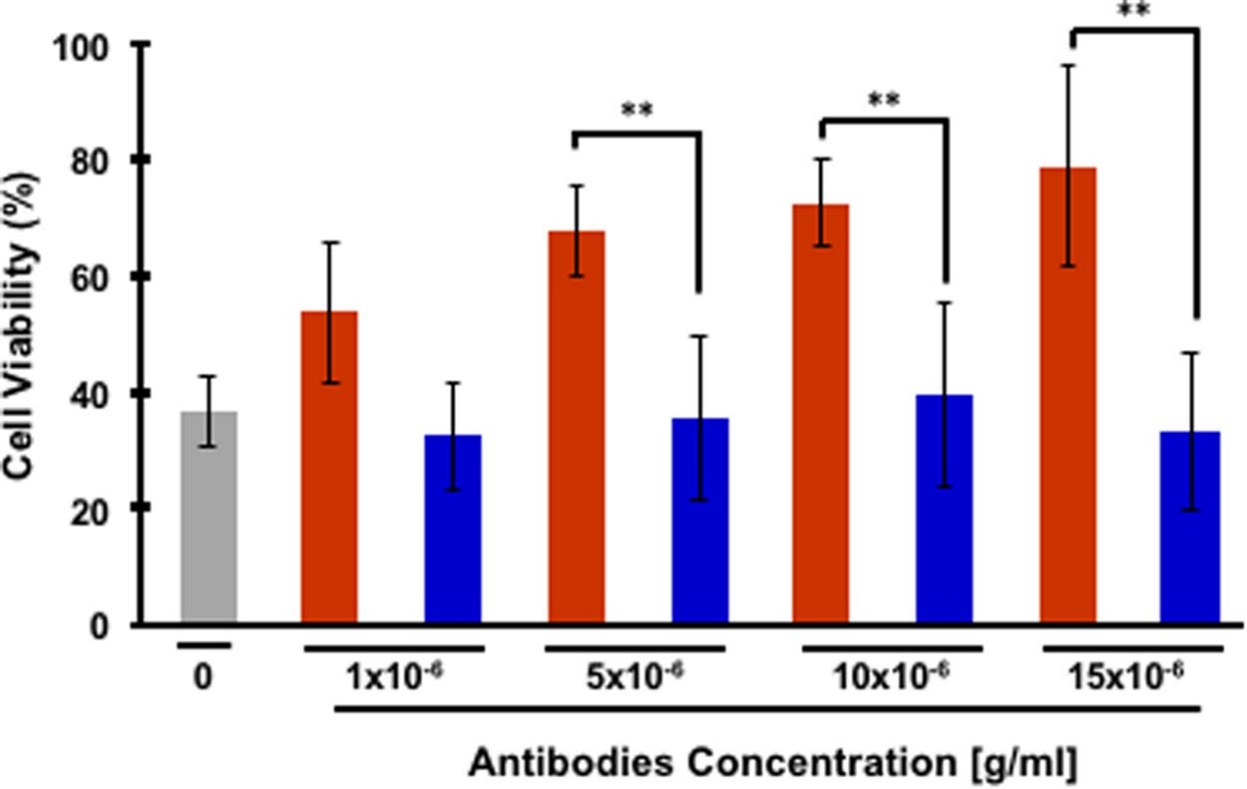
Antibodies derived from immunized mice recognize and neutralize hIAPP oligomers. Rin-m cells were treated with hIAPP oligomers with or without antibodies derived from the immunized (red) or the mock group (blue) at various concentrations. Cell viability was determined by the MTT reduction assay and treated and non-treated cells were compared (values are means ± s.d., Student’s t-test **P < 0.005).

Next, we wished to validate that the phenotype observed in the transgenic mice was the result of a direct interaction between the antibodies and hIAPP toxic conformers. Thus, we examined whether antibodies derived from the immunized mice or the mock group could reduce the cytotoxicity of hIAPP oligomers towards pancreatic β-cells. To this aim, β-cells were incubated with hIAPP oligomers and antibodies derived from the treated group or the mock group. As we and others previously reported, hIAPP oligomers are highly toxic^19,23,24^. Cells incubated with hIAPP oligomers exhibited a reduction in cellular viability of over 60%; while, the addition of antibodies derived from the immunized group resulted in a significant improvement in cell viability. As shown in Fig. 4, a dose-dependent increase in cellular viability, up to ~80%, was observed with an antibody concentration of 15 µg/ml. Importantly, the addition of antibodies derived from the mock group did not affect hIAPP cytotoxicity significantly.

As mentioned above, the appearance of amyloidogenic deposits in the histological sections of diabetic patients was first described more than a century ago^6^. Nevertheless, an effective non-symptomatic pharmacotherapy is still not available. In 2003, Charles Glabe’s group developed a conformation-dependent antibody targeting amyloid β (Aβ) oligomers and not monomeric or fibrillar aggregate conformations^25^ (A11 antibody). Interestingly, the antibody did not only bind Aβ oligomers but also recognized oligomers of other amyloidogenic peptides.

This important work discovered a common structural motif that amyloid protein share during the self-assembly cascade. In the following work, the same vaccination approach was used as an active immunization agent to target hIAPP self-assembly in the diabetes mice model^26^. Although high anti–hIAPP titers were observed, the treatment did not significantly influence β-cell mass reduction or delay the onset of the diabetes phenotype. A later study successfully determined for the first time the crystal structure αB-crystallin oligomers, a chaperone that can form amyloid fibrils^27^. The atomic structure of the oligomer revealed a β-sheet-rich structure organized in a cylindrical barrel; moreover, the αB-crystallin oligomers were recognized by the A11 antibody. As mentioned above, several studies have shown that hIAPP adopts an α-helical structure upon its interaction with biological membranes^28,29^. Gafni and coworkers demonstrated that hIAPP_1–19_ adopts a random coil conformation in solution and an α-helical conformation upon binding to lipid membranes, similar to the full-length peptide^30^. In addition, at low concentrations, the hIAPP_1–19_ fragment exhibits similar membrane permeabilization abilities; while, at high concentrations, the fragment induces a greater extent of membrane disruption than the full-length peptide and does not form amyloid fibrils like the wild-type sequence^30^. Furthermore, it was shown that the hIAPP_L12N/N14L_ variant is able to form β-sheet fibers but has limited capabilities to form α-helical structures; interestingly, this variant is significantly less toxic than the wild-type sequence^31^. All these data highlight the important role of early α-helical assemblies of hIAPP in the pathology of T2DM. Our results emphasize the importance of targeting the early α-helical oligomeric species rather than later β-sheet assemblies for inhibiting the pathological cascade that results in cell death and loss of pancreatic β-cell mass in T2DM. The results reported here also imply that the newly identified and characterized hIAPP species could serve as a valid epitope for the development of an active or passive vaccine targeting the late stages of T2DM.

## Methods

### Oligomers preparation

IAPP synthetic peptide (Human; H-7905, Bachem, Bubendorf, Switzerland) was dissolved in 100% 1, 1, 1, 3, 3, 3 hexafluoro-2-propanol (HFIP), 1 mg/ml and incubated for complete solubilization under shaking (100 rpm) at 37 °C for 2 hours. HFIP was removed by Speedvac apparatus (Eppendorf, Germany), the peptide was resuspended in 0.2 μM NaOH to a final concentration of 5 mM and sonicated for 2 minutes in a pre-chilled sonication bath to ensure complete solubilization. The peptide was diluted in phosphate-buffered saline (PBS, 10 mM pH 7.4) with 1% Sodium Dodecyl Sulfate (SDS) to a final concentration of 600 μM and incubated for 4–7 hours, 37 °C. Peptide was further diluted in ultra-pure H_2_O to a final concentration of 200 μM and incubated for 12 hours, 37 °C. IAPP self- assembly products were analyzed by 15% Tris-tricine PAGE and stained with Imperial protein stain. SDS was removed by using detergent removal spin column (Thermo-Fischer Scientific, U.S.A), samples were further dialyzed against PBS buffer overnight at 4 °C. IAPP oligomers were examined after treatment by PAGE analysis (non-reducing 15% Tris-tricine PAGE without boiling the sample) and size exclusion chromatography (Superdex 75) and shown no change in size distribution.

### Mice immunization

Homozygote mice FVB/N-Tg (Ins2-IAPP) RHFSoel/J (Jackson Laboratory) were intraperitoneally injected with hIAPP oligomers (100 μg) emulsified with Freund’s adjuvant (in a volume ratio of 1:1). The first injection was at the age of three weeks. Oligomers were mixed with complete Freund’s adjuvant (EMD MILLIPORE), followed by a boost in incomplete Freund’s adjuvant (EMD MILLIPORE) after 2 weeks, and monthly thereafter. hIAPP oligomers in PBS alone were injected from the fifth immunization onward.

All experimental and handling procedures were performed under the supervision and approval of the Tel Aviv University Institutional Animal Care and Use Committee (IAUCUC, m-11-079), in accordance with NIH guidelines for the humane treatment of animals.

### Glucose and insulin measurements

Blood glucose concentrations were examined after an 8 hour fast every 7 days. Values were measured from a tail-tip blood sample by a freestyle blood glucose meter (Accu-chek Performa). Following NIH guidelines for the humane treatment of animals, mice were sacrificed under anesthesia and the pancreas was removed. Insulin was extracted from pancreas by incubating the pancreas, diced into small fragments and homogenized, in 5 ml acid alcohol (1.5% HCl in 70% EtOH) at 4 °C for several days and neutralized with 1 M Tris buffer in a 1:1 ratio. Since the extraction is performed in acid alcohol the addition of protease inhibitors is not required. Insulin levels were determined using an ultrasensitive insulin ELISA kit (Mercodia).

### Antibodies recognition assay

Blood samples were collected by submandibular bleeding (N = 13). Antibodies serum titer was evaluated by ELISA assay. hIAPP oligomers (5 μg) were bound to Maleic Anhydride activated plates (Thermo-Fischer Scientific, U.S.A), 4 hour 37 °C. Wells were blocked with 3% bovine serum albumin (BSA) in PBS-T for 1 hour at room temperature; the plate was washed briefly with PBS and incubated with serum samples (in serial dilutions) for 2 hours at room temperature. ELISA plate was washed extensively with PBS-T and incubated with HRP-conjugated donkey anti-mouse HRP antibody (Jackson ImmunoResearch Laboratories, USA). Binding was quantified by 3, 3′, 5, 5′-tetramethylbenzidine reagent (TMB, Pierce) according to the manufacturer instruction.

### Tissue collection and analysis

Sedated mice were perfused through the heart with 20 ml of 4% paraformaldehyde, pancreas was removed in cold PBS, weighed and fixed in 4% paraformaldehyde, 4 °C 24 hours and embedded in paraffin. Histological sections (4 µm) were deparaffinized then washed with Tris-buffered saline (TBS)/0.1% Tween-20, then blocked with TBS/0.2% Triton X-100/3% BSA/2% normal donkey serum (Jackson Immunoresearch Laboratories, West Grove, PA) for 3 hours at room temperature. Sections were treated with human IAPP antibody (E-5, Santa Cruz Biotechnology) or Insulin antibody (H-86, Santa Cruz biotechnology). Images were acquired using LSM 510 Meta confocal laser scanning microscope (Carl Zeiss Jena, Germany).

### Antibodies purification

Antibodies from treated and mock groups were purified by protein-A/G column (GE healthcare). Serum was diluted 1:20 with loading buffer (20 mM Na_2_HPO_4_, 2 mM NaH_2_PO_4_ pH 7) and loaded onto a 5-ml protein-A/G column, flow throw was collected and reloaded 3 times. Bound antibody was eluted with 0.1 M of citric acid (pH 3.0) and neutralized with 1 M Tris-HCl (pH 9.0) for 1 ml of eluate, 200 μl of Tris buffer was added. Protein-containing fractions were combined, dialyzed against 2 liter PBS buffer (16 hours, 4 °C). Antibodies concentration was determined using Bradford reagent (Sigma-Aldrich).

### Antibodies neutralizing effect

Rin-m cells (2 × 10^5^ cells/ml) were cultured in 96-well microplates (100 μL/well) and incubated overnight at 37 °C. Human oligomers (5 μM, total peptide) were added to each well in the presence of the antibodies at various concentrations. Each measurement was repeated four times; also, a control measurement with antibodies alone at the highest concentration was performed to refute any effect of antibodies on cell viability. Following incubation for 6 hours at 37 °C, cell viability was evaluated using 3-(4, 5-dimethylthiazolyl-2)-2, 5-diphenyltetrazolium bromide (MTT) assay.

### Statistical Analysis

Quantitative results are shown as means ± SD. The statistical analysis was performed by Student’s t-test between control and tested groups. P value of ≤ 0.05 was considered significant. *Pv ≤ 0.05, **Pv ≤ 0.005 and ***Pv ≤ 0.0
